# Modified Approach of Manufacturer’s Power Curve Based on Improved Bins and K-Means++ Clustering

**DOI:** 10.3390/s22218133

**Published:** 2022-10-24

**Authors:** Yuan Fang, Yibo Wang, Chuang Liu, Guowei Cai

**Affiliations:** Key Laboratory of Modern Power System Simulation and Control and Renewable Energy Technology, Northeast Electric Power University, Jilin 132012, China

**Keywords:** wind turbine, manufacturer’s ideal power curve (MPC), practical power curve (PPC), Bins method, K-means++ clustering

## Abstract

The ideal wind turbine power curve provided by the manufacturer cannot monitor the practical performance of wind turbines accurately in the engineering stage; in this paper, a modified approach of the wind turbine power curve is proposed based on improved Bins and K-means++ clustering. By analyzing the wind speed-power data collected by the supervisory control and data acquisition system (SCADA), the relationship between wind speed and output is compared and elaborated on. On the basis of data preprocessing, an improved Bins method for equal frequency division of data is proposed, and the results are clustered through K-means++. Then, the wind turbine power curve correction is realized by data weighting and regression analysis. Finally, an example is given to show that the power curve of the same type of wind turbines, which, installed in different locations, are discrepant and different from the MPC, and the wind turbine power curve obtained by using this method can reflect the output characteristics of the wind turbine operating more effectively in a complex environment.

## 1. Introduction

In order to cope with the severe global energy crisis and prominent environmental problems, the development of renewable energy has attracted the attention of governments and organizations all over the world. Wind power, one of the most cost-effective forms of energy today, is emerging in the global energy market. According to the statistics of the Global Wind Energy Council (GWEC), in 2021, the newly installed capacity of wind power exceeds 93.6 GW, and the cumulative installed capacity reaches 837 GW [1]. With the increasing installed capacity, wind power companies pay more attention to the operation of wind turbines connected to the grid [2,3,4,5]. Among many factors reflecting the operating conditions, the power curve plays an important role and is an effective representation of the operating characteristics of wind turbines [6,7,8,9,10].

**Figure 1:**
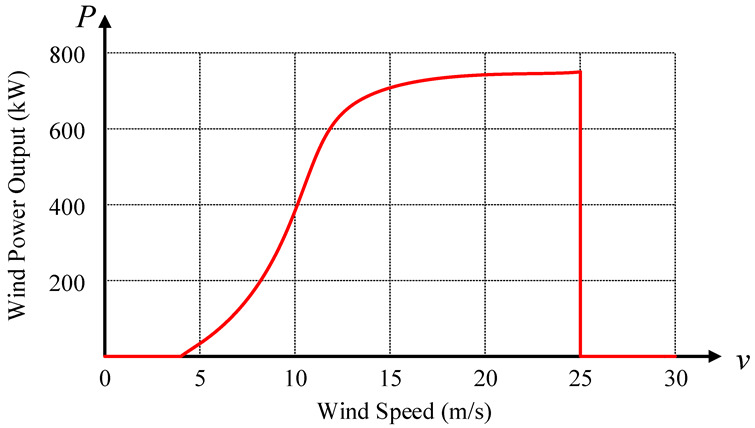
The manufacturer’s power curve (MPC).

The power curve of wind turbine shows the input-output relationship between wind speed and power from a macro perspective directly. As the power curve of wind turbine is an important indicator of generator performance, it affects the power generation capacity of wind turbine directly. Therefore, although the wind turbine acceptance standards of each wind power operation enterprise are different, the power curve is the content that all operation enterprises should check for consistency. Wind power equipment manufacturers will provide standard wind turbine power curves, i.e., manufacturer’s ideal power curve (MPC) as shown in Figure 1, that measured in “standard environment [11]” (15 C, 101.3 kPa) for each type of wind turbine when they leave the factory to reflect the output capacity of them.

**Figure 2:**
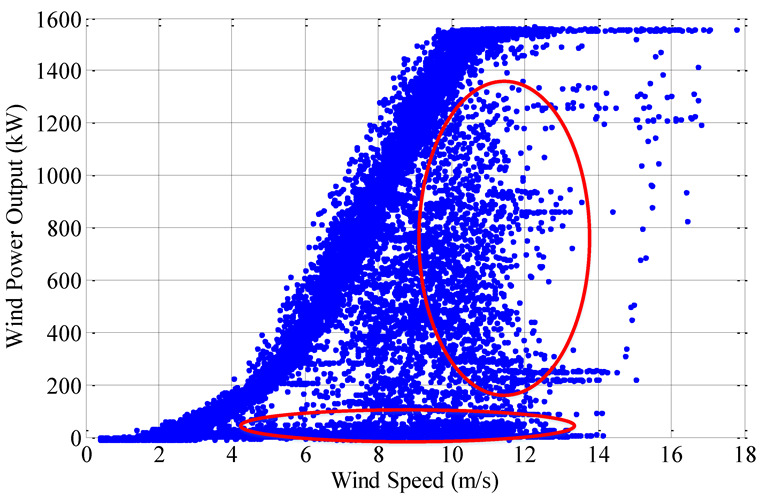
The scatter plot of a wind turbine.

However, the practical engineering operating environment of wind turbines installed in wind farms is much more complex than the “standard environment”, i.e., the output of wind turbines is affected by many factors, resulting in the fluctuation of output in a large range (Figure 2) rather than strictly in accordance with the MPC [12,13,14]. Considering the open and complex operating environment, the wind turbine has a relatively high outage probability. The outages of wind turbines are taken into account in [11], the relationship between the outage probability and wind speed is analyzed, and a model for including the outage probability of wind turbines in simulating the power output of wind farms is proposed. Different from [13], the power probability distribution functions (PPDFs) of each wind turbine are analyzed without considering the outages. Assuming that the wind turbines obey Poisson distribution in the statistical space of the wind farm, the wind speed-power characteristics of the wind farm are analyzed from the perspective of probability. In order to study the relationship between wind turbine output and wind speed, an artificial neural network method is used in [15,16] to estimate the wind power output based on wind information of meteorological tower, and then the wind speed-power curve is obtained. However, the model has six important parameters, and the selection of these parameters has a great influence on the accuracy of the model, so the generalization ability of the model is weak. Considering the different models for monitoring wind farms, a nonlinear parametric model is proposed in [17], and uses the wind speed as input to monitor the wind farm performance. However, from another point of view, it is unreasonable to use only a single wind speed power curve to characterize the output characteristics of wind turbines. Starting with the uncertainties of wind power curves, a confidence band is presented under test conditions [17]. 

For the problems mentioned above, modified approach of MPC based on improved Bins and K-means++ clustering is proposed in this paper, and the power curve obtained by this method can reflect the output characteristics of wind turbines in complex environments effectively. This paper is organized as follows. Section 2 reviews two representation methods of wind speed-power and data preprocessing. Section 3 describes the proposed modified approach based on improved Bins and K-means++ clustering. Section 4 presents the performance comparison based on simulated and real data of a wind farm. Section 5 concludes the paper.

## 2. Two Representation Methods and Data Preprocessing

According to mathematical statistics, there are two kinds of relations between random variables: functional relation and correlation. Similarly, the relations between wind speed and output of the wind turbine are displayed in two ways. The one is the functional relation expressed by power curve, e.g., MPC (Figure 1). The other is the correlation showed by scatter plot (Figure 2).

### 2.1. Functional Relation—Power Curve

According to the standard IEC 61400-12 [18], the wind speed-power characteristics of wind turbines can be expressed by wind speed-power curves. The curve reflects the relations between wind speed and the mean value of output in the time scale of 10 min, i.e., the mean value in 10 min is used as the object of data analysis to express the relations between them. This is a functional relation that represents the correspondence between wind speed and output. The power curve is shown in Figure 1.

As can be seen from Figure 1, a definite power value corresponds to each wind speed, i.e., the wind speed has a one-to-one correspondence with the output of the wind turbine. The cut-in and cut-out wind speed are 4 and 25 m/s, respectively. When the wind speed is between the two values, the output power of the wind turbine can be obtained according to the corresponding relations.

### 2.2. Correlation—Scatter Plot

Compared with the power curve, it is more practical to demonstrate the wind speed-power relationship by actual sampling data pairs (*v_i_*, *P_i_*) of wind turbines. As shown in Figure 2, it is a wind speed-power scatter plot with a time span of 3 months for a certain wind turbine at a sampling interval of 10 min.

From the sampled data, it can be seen that the wind speed-power relationship is not strictly following the power curve proposed by manufacturer based on actual engineering. Under a certain wind speed, the power value fluctuates in a wide range, i.e., the output is not unique, but a correlation.

### 2.3. Data Preprocessing

**Figure 3:**
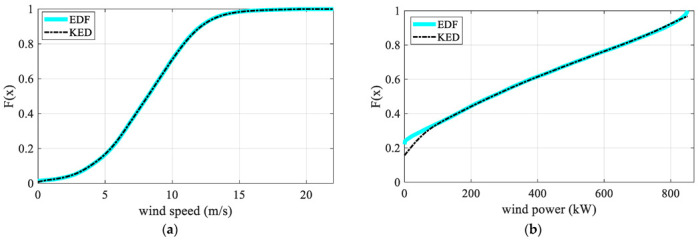
The empirical distribution function and kernel distribution estimation of wind speed and power: (**a**) the empirical distribution function and kernel distribution estimation of wind speed; (**b**) the empirical distribution function and kernel distribution estimation of wind power.

Because the data used to draw the scatter plot is sampled from wind turbines which are connected to the grid, the scatter plot is more authentic than the MPC in representing the operation characteristics of wind turbines. Therefore, the method adopted in this paper is also based on the actual sampling data of wind turbines. In order to understand the relationship between wind speed and output deeply, Copula theory is used. The non-parametric method (empirical distribution function (EDF) and kernel estimation method (KEM)) is used to analyze the sampled data, and then determine the overall distribution. The empirical distribution function diagram (EDFD) and kernel estimation diagram (KED) of wind speed and power are shown in Figure 3.

**Figure 4:**
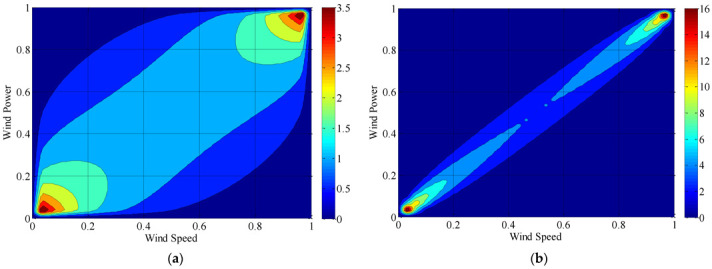
Distribution density map of wind speed and power: (**a**) distribution density map of wind speed; (**b**) distribution density map of wind power.

It can be seen from Figure 3 that the EDFD of wind speed and power almost coincide with the KED. Figure 4a shows the distribution density, and the binary normal Copula function Equation (1) and binary *t*-Copula function Equation (2) are selected to describe the correlation structure of the original data.

(1) $$C_{G}=\int_{-\infty }^{\Phi^{-1}(u)}\int_{-\infty }^{\Phi^{-1}(v)}\delta e^{\left\{-\frac{s^{2}-2\rho st+t^{2}}{2(1-\rho^{2})}\right\}}dsdt$$

(2) $$C_{t}=\int_{-\infty }^{t_{k}{}^{-1}(u)}\int_{-\infty }^{t_{k}{}^{-1}(v)}\delta {\left[1+\frac{s^{2}-2\rho st+t^{2}}{k(1-\rho^{2})}\right]}^{\frac{-(k+2)}{2}}dsdt$$

(3) $$\delta =\frac{1}{2\pi \sqrt{1-\rho^{2}}}$$

where *C_G_* is the binary normal Copula function and *C_t_* is the binary *t*-Copula function; Φ^−1^ is the inverse function of standard normal distribution function; *t_k_*^−1^ is the inverse function of a one-dimensional *t*-distribution function with *k* degrees of freedom; *ρ* is a second-order symmetric positive definite matrix with diagonal elements of 1.

In this paper, the rank correlation coefficients by Kendall and Spearman are used to study the correlation between wind speed and output. The correlation coefficient between wind speed and power is calculated based on the Copula function as shown in Table 1.

It can be seen from the calculation results that the correlation coefficients (Kendall and Spearman) between wind speed and power of the original sampling data are all below 0.65, meaning the correlation is weak. On the other hand, not all sampling points in the original sampling data set are reasonable and effective, such as the data points in the red circle in Figure 2. Regard such data points as sampling outliers, and using the method proposed in [10] to clean up them. Then, for the preprocessed data, the Copula theory is used again to calculate the correlation coefficient. The calculation results are shown in Figure 4b and Table 1. It can be seen that after data pretreatment, the correlation of measured data is improved. Compared with the original sample data, it is more reasonable to analyze the processed data, and the distribution density map shrinks to a smaller range, i.e., the data is more concentrated. Therefore, preprocessed data is selected for analysis in this paper.

## 3. Modified Approach Based on Improved Bins and K-Means++ Clustering

### 3.1. K-Means++ Clustering

In the process of solving practical engineering problems, clustering analysis is the process of grouping sampled samples into several classes composed of similar objects. Cluster analysis can classify characteristics of research objects or indicators by considering various factors, and according to the comprehensive properties of each sample, cluster analysis is completed. K-means++ [19,20,21] is a kind of “hard clustering” algorithm used in clustering analysis widely. Its algorithm idea and process are as follows.

Algorithmic idea: Assuming that *m* (0 < *m* < *K*) initial clustering centers have been selected, the point further away from the current *m* cluster centers has a higher probability of being selected as the *m* + 1th cluster center.

Algorithm process:

Step 1: A sample is selected from the sampled data set as the initial cluster center T_1_ randomly.

Step 2: The shortest distance *D*(*x*) between each sample and initial cluster center T_1_ is calculated.

Step 3: The probability *p*(*x*) of each sample being selected as the next cluster center is calculated. And the calculation information table of K-means++ as shown in Table 2.

(4) $$p(x)=\frac{D{\left(x\right)}^{2}}{\sum_{x\in X}D{\left(x\right)}^{2}}$$

Step 4: The next cluster center is obtained according to the roulette wheel method.

Step 5: Repeat Step 2 to Step 4 until all *K* clustering centers are selected.

Step 6: Calculate the distance from each sample to *K* clustering centers, select the nearest clustering center, and classify it into this category.

Step 7: Recalculate clustering center.

Step 8: Repeat Step 6 and Step 7 until there is no change in the class center.

### 3.2. Improved Bins

As mentioned above, the MPC is obtained based on the Bins method provided by IEC 61400-12. The Bins method takes wind speed as one of the research objects and divides the fluctuation range of wind speed into equal intervals, each of which is called a Bin. In the standard, the dividing interval of wind speed is 0.5 m/s, i.e., Bin size is 0.5, and all values of wind speed are integral multiple of 0.5 at each interval. Accordingly, the sampling wind speed interval can be divided into *n* Bins. On this basis, the average of wind speed and power in each Bin are calculated by Equations (5) and (6).

(5) $$v_{iav}=\frac{1}{n_{i}}\sum_{j=1}^{n_{i}}v_{ij}$$

(6) $$p_{iav}=\frac{1}{n_{i}}\sum_{j=1}^{n_{i}}p_{ij}$$

where *v_iav_* and *p_iav_* denote the average of wind speed and power in *i*th Bin, respectively; *n_i_* indicates the number of data in the *i*th Bin; (*v_ij_*, *p_ij_*) represents the wind speed and power of the *j*th sampling point in the *i*th Bin.

Using the measured data, a total of *n* two-dimensional data pairs (*v_iav_*, *p_iav_*) corresponding to each Bin are calculated by Equations (5) and (6). On this basis, the power curve based on the measured data and Bins method is obtained by interpolation method. The curve obtained by the above Bins method is limited by the value of Bin, the result obtained will be significantly different with the value of Bin changes, and no data is available in individual Bin, i.e., the Bin is empty. Therefore, when the curve obtained by this method is used to analyze the operating characteristics of the wind turbine, it will inevitably bring large errors. 

To solve the above problems, a correction method based on improved Bins and K-means++ clustering is proposed to modify the power curve. To ensure that there is sufficient sampling data in each Bin, on the basis of pretreatment of sampling data, the wind speed is divided into equal sampling points, i.e., equal frequency division. Each wind speed interval is denoted as one Bin, the entire wind speed range is divided into *n* Bins, and each Bin has the same sampling data. 

On this basis, clustering analysis is carried out for the sampled data in each Bin. In this paper, K-means++ clustering is adopted to process the data in Bins, and then, the weighted value of each category is calculated according to the weighted method.

(7) $$P_{ij}=\frac{1}{n_{ij}}\sum_{n=1}^{n_{ij}}P_{ij}(n)$$

(8) $$v_{ij}=\frac{1}{n_{ij}}\sum_{n=1}^{n_{ij}}v_{ij}(n)$$

(9) $$P_{i}=\left|\sqrt{\sum_{j=1}^{K}\left[\frac{n_{ij}}{n_{itotal}}{\left(P_{ij}\right)}^{2}\right]}\right|$$

(10) $$v_{i}=\left|\sqrt{\sum_{j=1}^{K}\left[\frac{n_{ij}}{n_{itotal}}{\left(v_{ij}\right)}^{2}\right]}\right|$$

where (*v_ij_*, *P_ij_*) represents the average wind speed and power of class *j* in Bin *i*. *n_ij_* represents the data volume of the *j*th class in the *i*th Bin; (*v_ij_* (*n*), *P_ij_* (*n*)) express the wind speed and power of the *n*th data point of the *j*th class in the *i*th Bin. (*v_i_*, *P_i_*) represents the equivalent power and wind speed in the *i*th Bin; *K* is the number of categories for clustering analysis.

**Figure 5:**
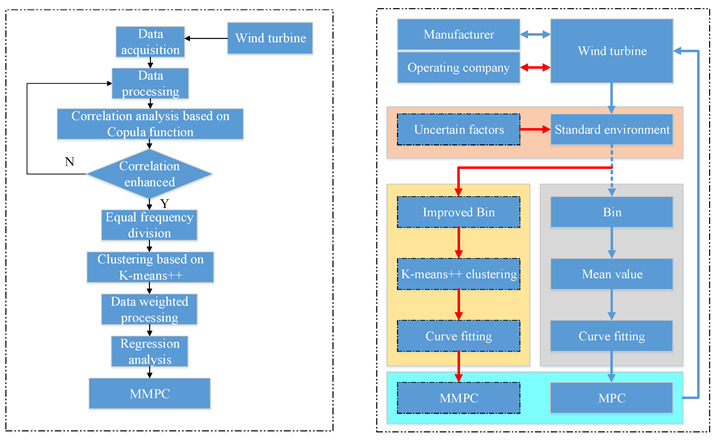
Flow chart of curve modify.

The *n_i_* data points used to express the performance of wind turbine are calculated by the above method. Then, the power curve is obtained by regression analysis. The curve optimization process is shown in the following figure (Figure 5).

## 4. Experiment and Results

### 4.1. Sample Data

**Figure 6:**
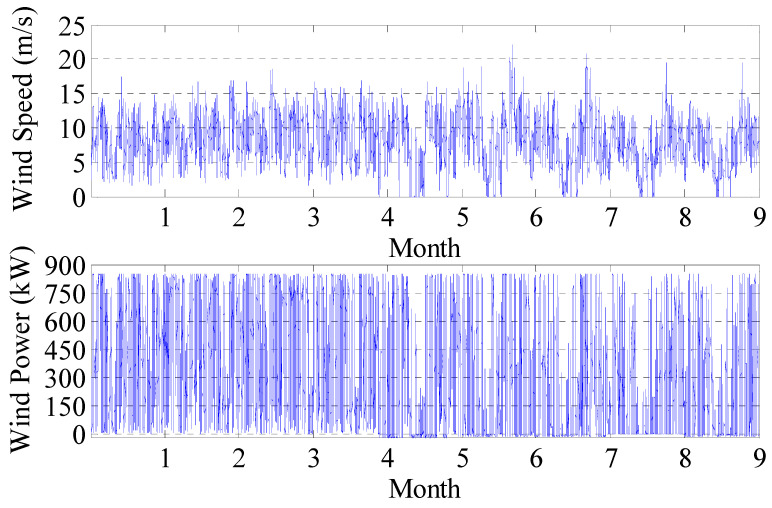
Schematic diagram of wind speed and power fluctuations.

**Figure 7:**
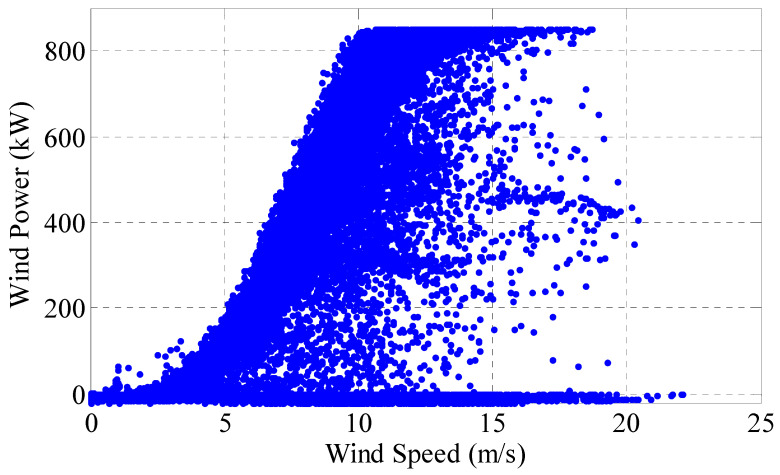
Wind speed-power scatter plot.

The wind turbine of a wind farm in Heilongjiang is selected randomly to verify the effectiveness of the proposed method. Meanwhile, the differences between the curve drawn by this method and MPC are compared and analyzed. The data are sampled in the SCADA system, and the sampling time and interval is 1 January 2017 solstice to 30 September 2017 and 10 min. The visualization effect of fluctuation and distribution of sampled data is as follows (Figure 6 and Figure 7).

### 4.2. Data Partitioning Based on Improved Bins

**Figure 8:**
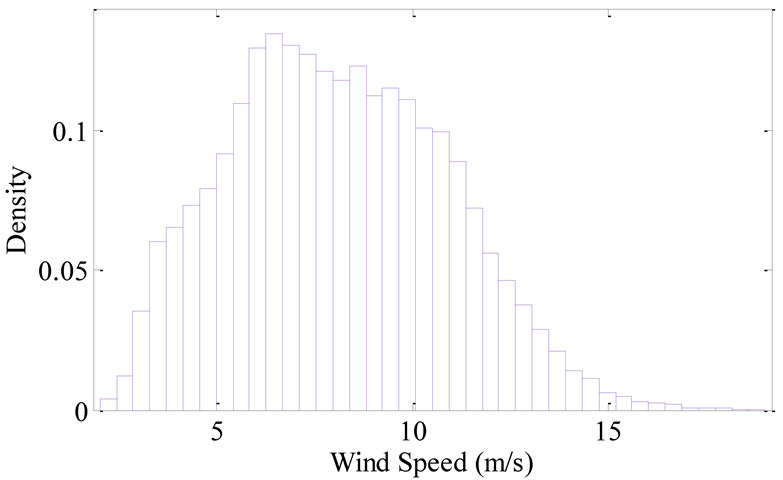
Wind speed frequency histogram.

**Figure 9:**
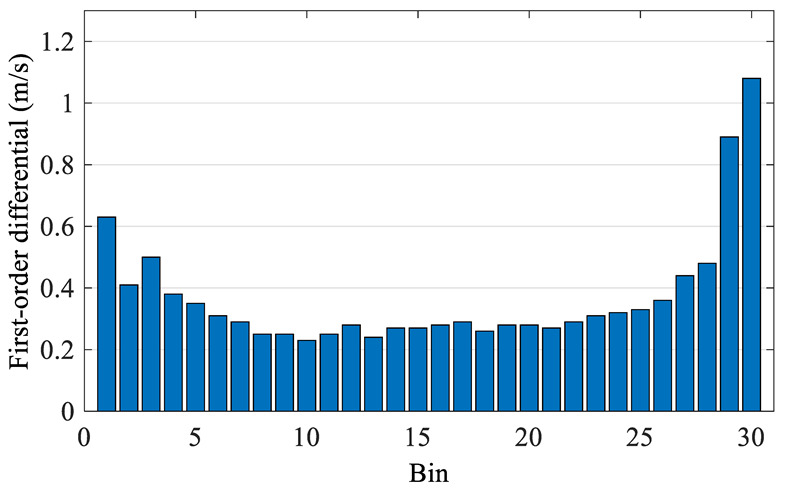
First-order differential histogram of wind speed based on improved Bin.

The raw data is preprocessed and wind speed frequency is plotted in a histogram (Figure 8). The preprocessed data are then divided by equal frequency using the improved Bins method proposed above. The first-order differential of each Bin is drawn in Figure 9.

As can be seen from the frequency histogram (Figure 8), the data size in each Bin is different especially in Bins at both ends. The improved Bins are equal-frequency division, i.e., the data amount in each Bin is equal. And in Figure 9 the first-order differential at both ends is larger than that in the middle, this phenomenon is caused by the different concentration of data in different wind sections. On the other hand, according to the mathematical statistics, it is more reasonable to study a certain problem by using different sample that has equal data. Therefore, the improved Bins method proposed in this paper is more reasonable.

### 4.3. Curve Correction of MPC

After the equal-frequency division of sampling data, K-means++ clustering algorithm is used to cluster the data in each Bin. Then, the data in each Bin are weighted by Equations (7)–(10), and the results are shown in Table 3.

**Figure 10:**
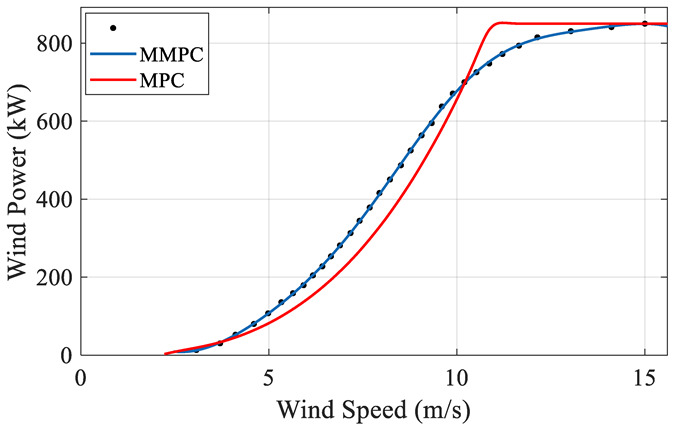
Wind speed-power curve fitting result.

Curve fitting based on the above data and compare the result with MPC in Figure 10.

As can be seen from the above diagram, the modified curve (MMPC) is different from that of MPC. When the wind speed is within [5 m/s, 10 m/s], the value of MMPC is greater than that of MPC, i.e., MMPC is above MPC. On the contrary, within the range [10 m/s, 15 m/s], MMPC is below the MPC. Since the MMPC is based on the measured data of wind turbine, it means that within the range [5 m/s, 10 m/s], the actual output is greater than the expected value. Within the range [10 m/s, 15 m/s], the actual output is less than the expected production value. Due to different results in different intervals, MMPC and MPC are quantitatively analyzed to compare the ability of both reflecting the real situation, as detailed below.

### 4.4. Contrastive Analysis

In the intervals of [5 m/s, 10 m/s] and [10 m/s, 15 m/s], the comparison results of MMPC and MPC are different (Figure 10), and the probabilities within the two intervals are different either, i.e., the data proportions are different. Under this scenario, the quantity of electricity calculated by MPC and MMPC are bound to be distinct. The two curves are compared and analyzed as follows.

MMPC and MPC are used to calculate daily power generation within 31 days.

**Figure 11:**
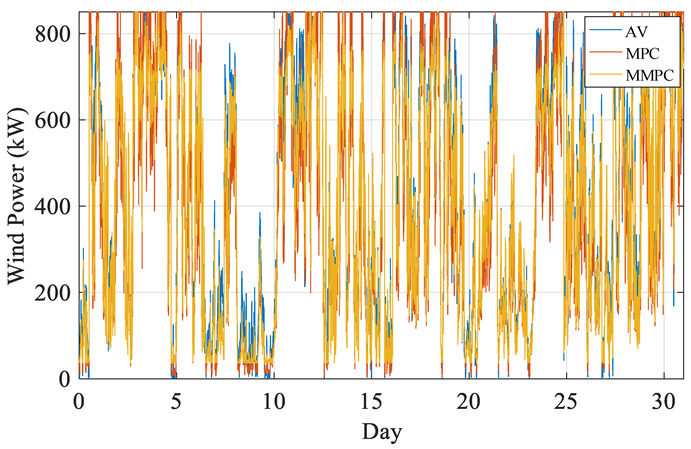
Comparison of wind power output during the month.

**Figure 12:**
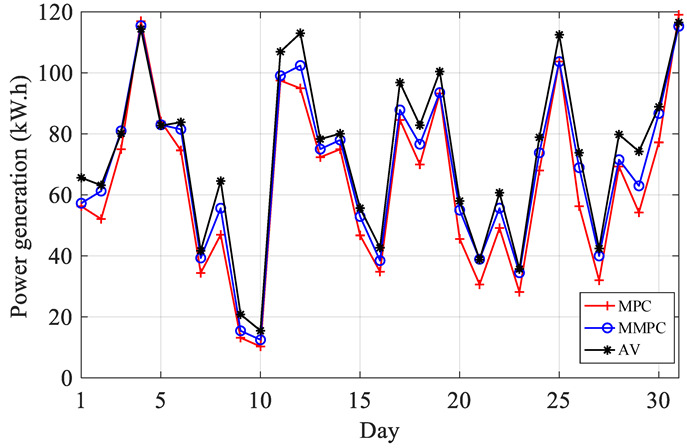
Comparison chart of Diurnal electric quantity.

The comparison chart of wind power output is shown in Figure 11. The quantity of electricity is expressed in Figure 12 and Table 4.

Where AV represents the actual value; MD denotes the deviation between the calculated value based on MPC and the actual value. MMD represents the deviation between the calculated value based on MMPC and the actual value. It can be seen from Table 4 that the modified result is all less than the value calculated by MPC, i.e., the result value is more accurate, and it can better reflect the actual operation of wind turbine.

## 5. Conclusions

The MPC of wind turbines is affected by many factors due to the complexity of the operating environment, e.g., the meteorological and environmental conditions at the installation of wind turbines, the operating state and operating life of wind turbines, etc., which make the relationship between the output of wind turbines and wind speed not strictly in accordance with MPC. Focus on the problem, based on the pretreatment of wind speed-power measured data of wind turbines sampled in engineering practice, a modified method is established based on improved Bin and K-means++ clustering in this paper. MMPC is obtained by this method, and the effectiveness of the proposed method is verified by an example analysis. The method proposed in this paper is helpful to express the output characteristics of wind turbines effectively connected to the network, and provides a certain reference for the manufacturers and management enterprises to have a deeper understanding of the operating characteristics of wind turbines. However, this method also has some limitations: due to the difference of wind turbine data sampling intervals, it will have a certain impact on the use of the method, which will be the content to be studied later.

## Figures and Tables

**Table 1:** Wind speed-power correlation coefficient.

Correlation Coefficient	Kendall		Spearman	
	Norm	*t*	Norm	*t*
Original data	0.4087	0.4717	0.5807	0.6391
Preprocessed data	0.8414	0.8556	0.9661	0.9689

**Table 2:** Calculation information table of K-means++.

Number of Samples	D (*x*)	D (*x*)^2^	*p* (*x*)	Sum
1	*d* (*x*_1,T1_)	*d* (*x*_1,T1_)^2^	*p* (*x*_1_)	*p* (*x*_1_)
2	*d* (*x*_2,T1_)	*d* (*x*_2,T1_)^2^	*p* (*x*_2_)	*p* (*x*_1_) + *p* (*x*_2_)
…	*d* (*x*)	*d* (*x*)^2^	*p* (*x*)	_∑_*p* (*x*_i_)
M	*d* (*x*_M,T1_)	*d* (*x*_M,T1_)^2^	*p* (*x*_M_)	1

**Table 3:** Data aggregation equivalent point.

Bin	Wind Speed	Power	Bin	Wind Speed	Power
1	3.07	13.24	17	8.22	450.38
2	3.70	30.29	18	8.51	486.84
3	4.11	52.40	19	8.77	524.89
4	4.60	80.06	20	9.06	563.43
5	4.98	107.39	21	9.33	595.08
6	5.33	135.78	22	9.60	637.65
7	5.64	158.82	23	9.89	670.90
8	5.92	179.31	24	10.20	699.96
9	6.17	204.60	25	10.52	725.47
10	6.42	227.73	26	10.86	747.77
11	6.65	253.55	27	11.21	772.13
12	6.89	281.42	28	11.65	793.66
13	7.17	313.03	29	12.14	814.99
14	7.41	344.46	30	13.03	830.77
15	7.68	378.50	31	14.11	841.22
16	7.94	415.85	32	15.01	850.00

**Table 4:** Comparison of Diurnal electric quantity (MW·h, %).

Date	MPC	MMPC	AV	MD	MMD
1	8.10	8.25	9.45	−14.29	−12.77
2	7.50	8.82	9.09	−17.52	−3.01
3	10.80	11.66	11.54	−6.45	1.04
4	16.84	16.63	16.47	2.26	0.97
5	12.11	11.95	11.91	1.64	0.29
6	10.74	11.74	12.07	−11.00	−2.76
7	4.96	5.66	6.01	−17.45	−5.72
8	6.76	8.01	9.29	−27.30	−13.79
9	1.90	2.23	3.00	−36.80	−25.81
10	1.48	1.80	2.23	−33.46	−19.18
11	14.04	14.27	15.39	−8.82	−7.34
12	13.67	14.75	16.27	−16.01	−9.39
13	10.42	10.80	11.27	−7.57	−4.19
14	10.79	11.23	11.53	−6.39	−2.59
15	6.73	7.62	8.01	−16.04	−4.91
16	5.02	5.54	6.14	−18.33	−9.82
17	12.18	12.65	13.94	−12.65	−9.26
18	10.08	11.03	11.92	−15.49	−7.47
19	13.42	13.47	14.46	−7.18	−6.88
20	6.56	7.92	8.35	−21.43	−5.18
21	4.41	5.60	5.58	−20.90	0.38
22	7.07	8.01	8.74	−19.11	−8.34
23	4.05	4.96	5.14	−21.08	−3.37
24	9.79	10.63	11.36	−13.81	−6.43
25	14.93	14.93	16.20	−7.81	−7.82
26	8.10	9.93	10.63	−23.79	−6.62
27	4.61	5.76	6.11	−24.58	−5.73
28	9.97	10.30	11.49	−13.19	−10.35
29	7.81	9.07	10.69	−26.93	−15.20
30	11.12	12.48	12.80	−13.11	−2.46
31	17.14	16.60	16.78	2.14	−1.09
Total	283.10	304.28	323.89	−12.59	−6.05
